# Quantitation of Diclofenac, Tolbutamide, and Warfarin as Typical CYP2C9 Substrates in Rat Plasma by UPLC-MS/MS and Its Application to Evaluate Linderane-Mediated Herb-Drug Interactions

**DOI:** 10.1155/2022/1900037

**Published:** 2022-03-10

**Authors:** Tingting Zhang, Ting Peng, Jinqiu Rao, Kai Wang, Feng Qiu

**Affiliations:** ^1^School of Chinese Materia Medica, Tianjin University of Traditional Chinese Medicine, Tianjin 301617, China; ^2^State Key Laboratory of Component-based Chinese Medicine, Tianjin University of Traditional Chinese Medicine, Tianjin 301617, China

## Abstract

Linderane (LDR), the main active and distinctive component of *L. aggregate*, is a mechanism-based inactivator of CYP2C9 *in vitro*, indicating the occurrence of herb-drug interactions. However, little is known about the changes of the pharmacokinetic properties of the common clinical drugs as CYP2C9 substrates after coadministration with LDR. In this study, a selective and rapid ultraperformance liquid chromatography-tandem mass spectrometry (UPLC-MS-MS) method for the determination of diclofenac, tolbutamide, and warfarin as CYP2C9 substrates in rat plasma has been developed. Chlorzoxazone was employed as an internal standard (IS), and protein precipitation was used for sample preparation. Chromatographic separation was achieved on a UPLC BEH-C_18_ (2.1 × 50 mm, 1.7 µm) with 0.1% (v:v) formic acid in water (*A*) and acetonitrile (B) as the mobile phase with gradient elution. The total run time was only 3.8 min. MS analysis was performed under multiple reaction monitoring (MRM) with electron spray ionization (ESI) operated in the negative mode. The bioanalytical method was validated, and the selectivity, carryover effects, linearity, precision, accuracy, matrix effect, extraction recovery, and stability were acceptable. The validated method was then successfully applied for evaluating the potential pharmacokinetic interactions when LDR was used along with diclofenac, tolbutamide, and warfarin, respectively. Results showed that the *C*_max_ of diclofenac in the treated group was 1287.82 ± 454.16 *μ*g/L, which was about 5-fold of that in the control group (*P* < 0.01). The *C*_max_ of tolbutamide in the treated group was 60.70 ± 10.70 mg/L, which was significantly decreased by about 25% when compared with the control group (*P* < 0.01). The *V*_d_ of warfarin in the treated group was obviously increased, which was about 1.4-fold of that in the control group (*P* < 0.01).

## 1. Introduction

**Figure 1:**
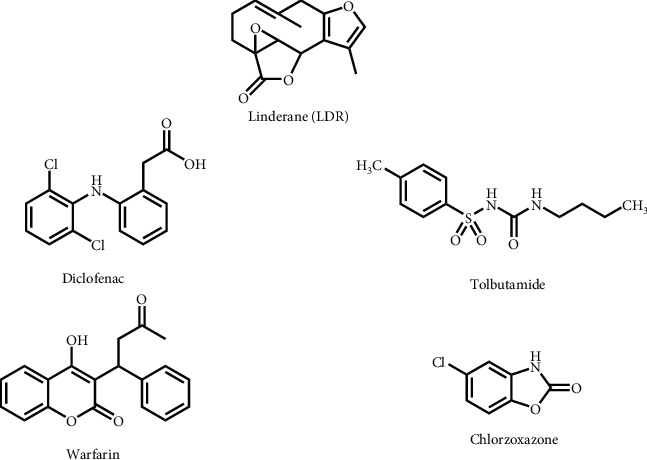
Structures of LDR, diclofenac, tolbutamide, warfarin, and chlorzoxazone.

*Lindera aggregate* (*L. aggregate*), derived from the root of *Lindera aggregate* (Sims) Kosterm, is usually found in the parts of southern China, Japan, and southeastern Asia [1]. It is usually used in traditional Chinese medicine as an analgesic and antispasmodic [2, 3]. *L. aggregate* was reported to have multiple pharmacological activities, such as decreasing sympathetic nerve activity to reduce blood pressure [4], protection of the central nervous system [5, 6], slowing down the progression of diabetic nephropathy [7], and antitumor [8, 9]. Sesquiterpenes, alkaloids, and tannins have been documented as the main components of *L. aggregate* [10]. Linderane (LDR, Figure 1), which belongs to furan-containing sesquiterpene lactone, is one of the main active components of *L. aggregate* [11]. Based on the fact that the furan ring of LDR is often regarded as a “structural alert” that may trigger the inhibition of CYP450 enzymes (P450 enzymes) or produce organ toxicity [12], we previously proved that LDR could irreversibly inhibit the activity of CYP2C9 *in vitro* and the inhibition was (nicotinamide adenine dinucleotide phosphate) NADPH-, time-, and dose-dependent [13]. The inactivation of CYP2C9 caused by LDR was related to the electrophilic reactive intermediates produced by the metabolic activation of the furan ring of LDR. Hence, LDR was proved to be a mechanism-based inactivator of CYP2C9.

CYP2C9 is one of the most abundant P450 enzyme subtypes, accounting for about 20% of the P450 enzymes. About 15% of the commonly used clinical drugs, such as tolbutamide, warfarin, diclofenac, and losartan, are mainly metabolized by CYP2C9 [14]. Studies have shown that once a drug inhibits the activities of P450 enzymes, it may affect the pharmacokinetic behaviors of the substrates of the corresponding enzyme subtypes, resulting in drug accumulation, even severe adverse reactions [15, 16]. Mechanism-based inactivators could specifically inhibit the activities of P450 enzymes. Hence, if CYP2C9 is inactivated in a mechanism-based manner, serious herb-drug interactions (HDIs) may occur, especially for the substrates with narrow therapeutic windows, such as warfarin, a common anticoagulant in the clinic.

Many natural compounds could inhibit the activity of CYP2C9 and may further trigger drug-drug interactions (DDIs) *in vivo* [17]. Piperine, the bioactive compound of black pepper, could affect the pharmacokinetic behavior of 7-hydroxywarfarin, which is the main metabolite of warfarin catalyzed by CYP2C9. Piperine could inhibit warfarin clearance and reduce the plasma concentration of 7-hydroxywarfarin by inhibiting the activity of CYP2C9 [18]. Similarly, a retrospective cohort study showed that a total of 22,272 veterans were treated with antibiotics after warfarin treatment. About 8194 patients were treated with low-risk antibiotics, such as trimethoprim/sulfamethoxazole (TMP/SMX), ciprofloxacin, and levofloxacin, and 14,078 patients were treated with high-risk antibiotics, such as clindamycin and cephalexin. Unfortunately, about 129 severe bleeding events were reported in the two groups [19]. In addition, honokiol, the main component of *Magnolia officinalis*, could significantly increase the *t*_1/2_ and AUC of tolbutamide, mainly because of the inhibitory effect of honokiol on the activity of CYP2C9 [20]. It is obvious that DDIs based on the inhibition of the activity of CYP2C9 are frequently reported. As traditional Chinese medicine is widely used in clinical practice, the studies on HDIs caused by some natural compounds are of vital importance for guiding the rational use of drugs and predicting adverse reactions. Since the irreversible inhibition of CYP2C9 by LDR has only been confirmed *in vitro*, it is not clear whether LDR could also trigger DDIs by irreversibly inhibiting CYP2C9 *in vivo*.

In this study, LDR was used along with the three classical substrates of CYP2C9, namely diclofenac, tolbutamide, and warfarin, to explore whether LDR would affect the pharmacokinetic behaviors of these clinical drugs. Currently, most methods of the sample preparations of these three substrates in published literature were liquid–liquid extraction (LLE), which was cumbersome and time-consuming [21, 22]. In addition, the equipment applied in these researches has relatively low sensitivity [23–26]. To improve the analytical methods, a selective and rapid method for the quantification of diclofenac, tolbutamide, and warfarin in rat plasma was established first. Then, the validated method was successfully applied to the pharmacokinetic studies of the three substrates after coadministration with LDR in rats. The objective of the study was to provide a simple, reliable, and rapid method for predicting CYP2C9-mediated DDIs in the preclinical study.

## 2. Materials and Methods

### 2.1. Chemicals and Reagents

LDR (purity >98%), diclofenac (purity >99%), tolbutamide (purity >99%), warfarin (purity >98%), and chlorzoxazone (purity >98%) were obtained from Shanghai Yuanye Biological Technology Co., Ltd. (Shanghai, China). Sodium carboxyl methyl cellulose (CMC-Na) was purchased from Tianjin Damao Chemical Reagent Factory (Tianjin, China). Ethylenediaminetetraacetic acid disodium salt (EDTA-Na_2_) was purchased from Beijing Solarbio Science and Technology Co., Ltd. (Beijing, China). Methanol and acetonitrile of LC-MS grade were obtained from Sigma-Aldrich (St. Louis, MO). Formic acid of LC-MS grade was purchased from Shanghai Macklin Biochemical Co., Ltd. (Shanghai, China).

### 2.2. LC-MS/MS Method

Chromatographic separation was performed on an ACQUITY UPLC BEH-C_18_ (2.1 × 50 mm, 1.7 µm) with a mobile phase of (*A*) 0.1% (v: v) formic acid in water and (B) acetonitrile at a flow rate of 0.3 mL/min with gradient elution (0–2.0 min, 10% B-95% B; 2.0–2.8 min, 95% B; 2.8–3.0 min, 95% B-10% B; 3.0–3.8 min, 10% B). The column was set at indoor temperature, and the sample injection volume was 2 µL for analysis.

For the determination of the pharmacokinetic behavior of diclofenac, the UPLC-MS/MS analysis was performed with a Xevo TQ-XS (Waters Corporation, Milford, USA) triple quadrupole mass spectrometer with the instrumental parameters as follows: source gas flow at 550 L/Hr, cone flow at 10 L/hr, desolvation temperature at 600 °C, cone voltage at 20 V, and capillary voltage of 0.8 kV. The optimized mass parameters for the detection of diclofenac and IS with negative ion mode are shown in Table 1. Data acquisition was controlled using the MassLynx software.

For the determination of the pharmacokinetic behaviors of tolbutamide and warfarin, the UPLC-MS/MS analysis was performed with AB SCIEX 5500 triple quadrupole mass spectrometer under the multiple reaction monitoring (MRM) mode. The instrumental conditions were set as follows: curtain gas (CUR): 30.00 psi; collision gas (CAD): 10.00 psi; ion spray voltage (IV): −4500.00 V; temperature: 500.00 °C; ion source gas1 (GS1): 50.00 psi; ion source gas2 (GS2): 50.00 psi. The mass scan method parameters of tolbutamide, warfarin, and IS are shown in Table 2. Data acquisition was controlled using the Analyst 1.6.2 software.

### 2.3. Animals

All animal experiments were approved by the Animal Ethics Committee of Tianjin University of Traditional Chinese Medicine and conducted on the basis of the Guide for the Care and Use of Laboratory Animals by the National Institutes of Health of China. Male Sprague-Dawley (SD) rats (200–220 g) were obtained from the SPF Biotechnology Co., Ltd. (Beijing, China). The experimental animals had free access to water and food. They were maintained for 5 days on standard rat chow in a controlled environment at room temperature (20–25 °C) and moderate humidity (50%–70%), under 12 h dark/light cycles.

### 2.4. Pharmacokinetic Studies

Thirty-six male Sprague Dawley (SD) rats were randomly divided into the control group and treated group with eighteen rats in each group. The two groups were intragastrically given 20 mg/kg of LDR or an identical volume of 0.5% CMC-Na for fifteen consecutive days, respectively. Every six rats in the control group or treated group were intragastrically given 2.0 mg/kg diclofenac, 30.0 mg/kg tolbutamide, and 2.0 mg/kg warfarin, respectively, on the sixteenth day. The blood sample was collected into EDTA-Na_2_ centrifuge tubes at 0.080, 0.25, 0.50, 1.0, 2.0, 4.0, 8.0, 12, 24 h, 48 h, 72 h, and 96 h after administration, respectively. After centrifugation at 8,000 rpm for 10 min, the supernatant was transferred and stored at −80 °C before analysis.

### 2.5. Plasma Sample Preparation

All frozen rat plasma samples were thawed at room temperature and vortex-mixed. For the analysis of the concentration of diclofenac or warfarin in plasma, an aliquot of 20 µL rat plasma and 20 µL of 50% acetonitrile were vortex-mixed for 1 min. The samples were processed with 200 µL of acetonitrile solution containing chlorzoxazone (IS, 500 ng/mL) to denature the protein, and they were vortex-mixed for 2 min. After centrifugation at 14,000 rpm for 10 min, 150 µL of the supernatant layer was transferred, followed by the addition of 50 *μ*L of 50% acetonitrile. The resulting mixture was vortex-mixed and centrifuged with the supernatant for UPLC-MS/MS analysis.

For the analysis of the concentration of tolbutamide in plasma, an aliquot of 10 µL of rat plasma and 10 µL of 50% acetonitrile were added into a centrifuge tube and vortexed for 1 min. The samples were processed with 200 µL of acetonitrile solution containing chlorzoxazone (IS, 500 ng/mL) to denature the protein. Then, the mixture was vortexed for 2 min. After centrifugation at 14,000 rpm for 10 min, 10 µL of the supernatant layer was transferred into another clean tube and 990 *μ*L of 50% acetonitrile was added and then vortexed for 1 min, followed by centrifugation at 14,000 rpm for 10 min. A 2 *μ*L aliquot of the supernatant was taken for UPLC-MS/MS analysis.

### 2.6. Preparation of Plasma Standards and Quality Control Samples

The standard stock solutions of diclofenac, tolbutamide, and warfarin (1.0 mg/mL) were prepared in methanol solution. The working solutions for calibration and controls were subsequently prepared by appropriate dilution in 50% methanol solution. The spiked plasma samples at all the levels were stored at −20°C for validation and subject sample analysis.

### 2.7. Statistical Analysis

Pharmacokinetics analysis was performed using a noncompartmental model by the Drug and Statistic version 3.2.8 (DAS 3.2.8) software program to calculate the pharmacokinetic parameters, such as *T*_max_, *C*_max_, AUC_0-t_, AUC_0-∞_, *V*_d_, and CL. All of the data were expressed as‾*x* ± *s*. SPSS 26.0 software was used for statistical data, and the *t*-test was used for comparison between groups.

## 3. Results and Discussion

### 3.1. Method Development and Optimization

UPLC-MS/MS was employed to detect the plasma concentrations of the three substrates of CYP2C9. The application of triple quadrupole mass spectrometry has led to higher detection sensitivity and lower detection limit compared with the equipment reported in other literature [23–26]. As for the determination of diclofenac, Waters Xevo TQ-XS triple quadrupole mass spectrometer showed higher sensitivity compared with AB SCIEX 5500 triple quadrupole mass spectrometer. We selected the negative ion mode for detecting the three substrates and IS because of their higher response. The optimal product ions were then confirmed and relevant mass parameters were optimized (Tables 1 and 2). Chromatographic separation was achieved on a UPLC BEH-C_18_ (2.1 × 50 mm, 1.7 µm) with 0.1% (v: v) formic acid in water (A) and acetonitrile (B) as the mobile phase with gradient elution. The total run time was only 3.8 min, which was more rapid than the methods that have been published [27–30]. The plasma samples were extracted by protein precipitation, which is simple, convenient, and time-saving compared with the liquid-liquid extraction methods reported in the literature [21, 22].

### 3.2. Method Validation

#### 3.2.1. Specificity and Carryover Effects

**Figure 2:**
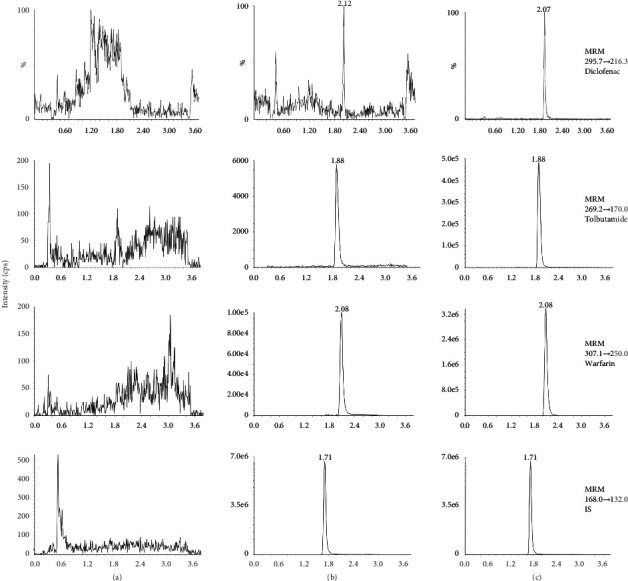
MRM chromatograms of diclofenac, tolbutamide, warfarin and chlorzoxazone (IS) from rat plasma. (a) Blank rat plasma; (b) Blank plasma samples spiked with standard substance (LLOQ) and IS; (c) Real plasma samples obtained from a rat following administration of 2 mg/kg diclofenac, 30 mg/kg tolbutamide, and 2 mg/kg warfarin.

The specificity of the LC-MS/MS method was investigated by analyzing blank rat plasma from six different sources to ensure that there were no interfering peaks at the retention time of diclofenac, tolbutamide, warfarin, and IS. All six blank plasma samples were free of interfering signals at the retention time of diclofenac, tolbutamide, warfarin, and IS. Thus, the method was specific for diclofenac, tolbutamide, and warfarin determination. Figures 2(a)–2(c) are the chromatograms of blank rat plasma, blank plasma samples spiked with standard substance (LLOQ), and real plasma samples obtained from a rat following the administration of 2 mg/kg diclofenac, 30 mg/kg tolbutamide, and 2 mg/kg warfarin, respectively. Carryover was assessed by the two consecutive injections of an extracted blank sample after the injection of an extracted upper limit of quantitation (ULOQ) sample. No peak of the analyte or IS from the blank sample was observed, indicating no carryover from residues in the autosampler.

#### 3.2.2. Linearity, Precision, and Accuracy

The linearity was checked by analyzing the duplicate of calibration standards for three consecutive runs. The calibration curve was plotted by the area ratio (*y*) of analyte/IS obtained from MRM versus the nominal concentration (*x*). Each calibration curve was analyzed individually using least square weighted (1/*x*^2^) linear regression. The calibration curves were found to be linear over the specified concentration range of 0.020–5.0 *μ*g/mL for diclofenac, with a correlation coefficient (*r*) of 0.9998, 0.200–100 *μ*g/mL for tolbutamide, with a correlation coefficient (*r*) of 0.9973, and 0.050–20 *μ*g/mL for warfarin, with a correlation coefficient (*r*) of 0.9977. The LLOQ were 0.020 *μ*g/mL for diclofenac, 0.200 *μ*g/mL for tolbutamide, and 0.050 *μ*g/mL for warfarin, which were proved to be sufficient for the pharmacokinetic studies in rats (Table 3). The precision and accuracy data for the determination of diclofenac, tolbutamide, and warfarin at three QC levels are summarized in Table 4. The intraday and interday precisions for all the analytes ranged from 0.82% to 5.76% and 2.02% to 6.27%. The RE was between -6.9% and 10.3%. The results of the intraday and interday precision and accuracy studies were well within the acceptable limits (Table 4).

#### 3.2.3. Matrix Effect and Extraction Recovery

The matrix effects were determined by calculating the ratio of the peak area in the presence of matrix (measured by analyzing the blank matrix spiked with the analyte or IS) to the peak area in the absence of matrix (pure solution of the analyte or IS). The extraction recovery was determined by comparing the peak area of analyte or IS obtained from plasma samples with the analyte or IS spiked before extraction with that spiked after extraction. The results of recovery and matrix effect were listed in Table 4.

#### 3.2.4. Stability

The freeze-thaw stability, long-term stability, short-term stability, and postpreparative stability results in plasma samples at two QC concentration levels are shown in Table 5. The RSD values ranged from 0.5% to 7.4%. These results indicated that diclofenac, tolbutamide, and warfarin were stable in rat plasma under the test conditions.

### 3.3. Pharmacokinetic Application and Discussion

In this study, the UPLC-MS/MS method was applied to investigate how the pharmacokinetic behaviors of the three commonly used substrates of CYP2C9 in male SD rats were changed after intragastrically giving 20 mg/kg of LDR for fifteen consecutive days. We established a rapid and selective method that required only a small quantity of rat plasma. In addition, plasma samples were processed using a simple and time-saving method of protein precipitation.

#### 3.3.1. Influence on Pharmacokinetic Profiles of Diclofenac in Rats by LDR

**Figure 3:**
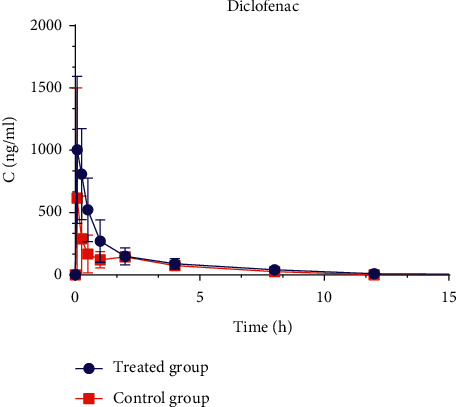
Plasma concentration–time curves of diclofenac in treated group and control group. Six rats in each group were intragastrically given 20 mg/kg of LDR (treated group) or identical volume of 0.5% CMC-Na (control group) for fifteen consecutive days, respectively. The two groups were intragastrically given diclofenac prepared in 0.5% CMC-Na at a dose of 2 mg/kg at the sixteenth day.

In the experiment that explored whether LDR could affect the pharmacokinetic behavior of diclofenac, a nonsteroidal anti-inflammatory drug (Figure 1), consistent plasma concentration-time curves *in vivo* were observed compared with the results of enzyme inhibition *in vitro* (Figure 3). Diclofenac was almost undetectable after 12 h in both control and treated groups. For rats in the treated group with the pretreatment of LDR, the parameter of *C*_max_ was 1287.82 ± 454.16 *μ*g/L, about 5 times compared with the control group (*P* < 0.01). The values of AUC_0-t_ and AUC_0-∞_ were significantly increased by about 1-fold compared with the control group (*P* < 0.01), while *V*_d_ and CL were significantly decreased by about 50% compared with the control group, which means diclofenac was markedly accumulated in the rats of the treated group (Table 6). The results proved that LDR could indeed significantly inhibit the enzyme activity after the mechanism-based inactivation of CYP2C9, thus inhibiting the metabolism of diclofenac and increasing its plasma concentration *in vivo*.

Furthermore, another study has shown that quercetin could affect the pharmacokinetic behavior of diclofenac in healthy volunteers. The parameters of *C*_max_ and AUC_0-∞_ in the quercetin group were about 2-fold compared with the control group [31]. The results suggested that quercetin might have inhibited the CYP2C9-mediated metabolism of diclofenac. Similarly, the *C*_max_ of diclofenac in the treated group was about 5-fold of that in the control group after intragastrically giving 20 mg/kg of LDR for fifteen consecutive days. Thus, there is a potential pharmacokinetic interaction present between LDR and diclofenac. Accordingly, caution should be taken when *L. aggregate* is used in combination with diclofenac.

#### 3.3.2. Influence on Pharmacokinetic Profiles of Tolbutamide in Rats by LDR

**Figure 4:**
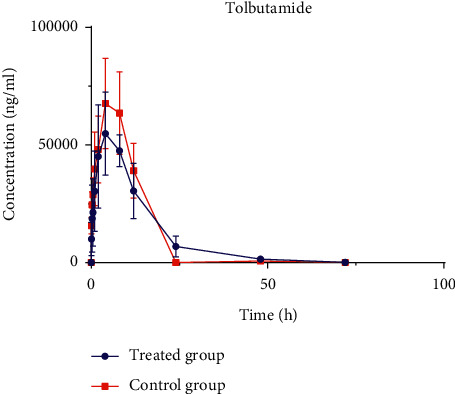
Plasma concentration–time curves of tolbutamide in treated group and control group. Six rats in each group were intragastrically given 20 mg/kg of LDR (treated group) or identical volume of 0.5% CMC-Na (control group) for fifteen consecutive days, respectively. The two groups were intragastrically given tolbutamide prepared in 0.5% CMC-Na at a dose of 30 mg/kg at the sixteenth day.

Contrary to our expectation, the results in this experiment (Figure 4 and Table 7) were perfectly inconsistent with the phenomena of enzyme inhibition *in vitro*. The *C*_max_ of rats in the treated group was 60.70 ± 10.70 mg/L, which was significantly decreased by about 25% compared with the control group (*P* < 0.01). The parameters of AUC_0-t_ and AUC_0-∞_ in the treated groups were lower than those in the control groups, which indicated that the absorption of these two substrates may be weakened after being used along with LDR.

Tolbutamide, as the first generation of sulfonylurea oral hypoglycemic drug (Figure 1), mainly acts selectively on the pancreatic *β* cells to promote insulin secretion, thus reducing blood glucose [32, 33]. Tolbutamide is mainly metabolized by CYP2C9 in the liver. Hence, it is often applied as a substrate of CYP2C9 to explore the changes of enzyme activity [20]. The results of the previous studies showed that LDR could irreversibly inhibit the enzyme activity after being inactivated because of the mechanism-based activity of CYP2C9 *in vitro*. However, compared with the control group, the *C*_max_ of tolbutamide in the treated group was significantly decreased. The absorption, distribution, metabolism, and excretion of drugs *in vivo* are more complex than those *in vitro*. In the earlier stage of this study, the time-dependent inhibition of LDR on different P450 enzyme subtypes in the liver microsomal incubations alone was investigated, while the impact of LDR on some drug transporters has not been explored. Additionally, some studies also found that there was a significant difference in the CL of tolbutamide *in vivo* and *in vitro* [34, 35]. Therefore, we hypothesized that the presence of some drug transporters in the rats affects the metabolism of tolbutamide by affecting CL. Researchers proved that liver organic anion transporter 2 (OAT2)-mediated uptake for the liver cells would also affect the plasma concentration of tolbutamide [36]. Therefore, the pharmacokinetic behavior of tolbutamide in rats was influenced by OAT2 and CYP2C9. Hence, we conjectured that LDR may promote the uptake transporter OAT2 in the liver so that more tolbutamide could be absorbed into the liver cells, thereby increasing CL and reducing plasma concentration. In addition, verification experiments about the specific mechanisms are underway.

#### 3.3.3. Influence on Pharmacokinetic Profiles of Warfarin in Rats by LDR

**Figure 5:**
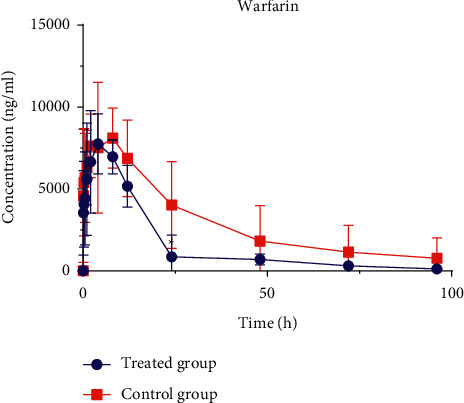
Plasma concentration–time curves of warfarin in treated group and control group. Six rats in each group were intragastrically given 20 mg/kg of LDR (treated group) or identical volume of 0.5% CMC-Na (control group) for fifteen consecutive days, respectively. The two groups were intragastrically given warfarin prepared in 0.5% CMC-Na at a dose of 2 mg/kg at the sixteenth day.

The same phenomena were observed in another set of experiments exploring whether LDR affected the pharmacokinetic behavior of warfarin in rats. The mean plasma concentration-time curves of warfarin in the control group and the treated group (Figure 5) were determined by the above method. The main pharmacokinetic parameters of warfarin are shown in Table 8. *V*_d_ of rats in the treated group was significantly increased (about 1.4 times, (*P* < 0.01)) compared with that in the control group. The parameters of *t*_1/2_, AUC_0-t_, and AUC_0-∞_ showed a decreasing trend compared with the control group, however, there was no statistical difference.

Although many studies have reported that warfarin (Figure 1) was prone to DDIs because of its narrow therapeutic window, it is still the first choice for long-term clinical anticoagulation [37]. Warfarin is given to patients as a racemic mixture consisting of equal amounts of the R- and S-enantiomers, and the potency of S-warfarin, which is mainly metabolized by CYP2C9, is much higher than that of R-warfarin [38]. The pharmacokinetic studies show that the plasma protein binding rate of warfarin is 99.4%. Warfarin should not be used with drugs with a high plasma protein binding rate. Otherwise, its anticoagulation effect will be enhanced. we take into account a previous piece of research, in which it was shown that piperine [39], a potent inhibitor of CYP2C9, for its high lipophilicity, is able to influence the bioavailability of warfarin, even with a binding displacement mechanism. We speculated that LDR, a very lipophilic molecule for its unique lactone structure, after being intragastrically administered (20 mg/kg) for fifteen consecutively days, may compete and replace warfarin that is bound to plasma proteins. Thus, the plasma protein-bound concentration (C_bound_) of warfarin probably decreased, resulting in the reduction of C_total_ (which is the sum of free concentration (C_free_) and C_bound_), while the free drug fraction (fu), given by C_free_/C_total_, increased. However, it is worth noting that an increase of free drug fraction is not necessarily associated with an increase in C_free_. Unlike the case of *in vitro*, C_total_ is not fixed, and drug displacement from proteins may not change C_free_*in vivo*, however, the results are in a reduction in C_total_ [40], thus increasing fu. Once the C_total_ of warfarin decreases, *V*_d_ increases.

In addition, it has also been reported that OAT2 is involved in the clearance process of warfarin, which may cause individual differences [41]. As speculated above, once LDR promoted OAT2, more warfarin would be taken up into the liver cells, and the plasma concentration would reduce, however, the detailed mechanisms still need further exploration.

## 4. Conclusion

In this study, we developed a more rapid UPLC-MS/MS method, which was set for the determination of diclofenac, tolbutamide, and warfarin in rat plasma for evaluating the effects of LDR on the pharmacokinetic behaviors of the three classic substrates of CYP2C9 to verify CYP2C9 inhibition *in vitro*. The results showed that the pharmacokinetic behavior of diclofenac was consistent with the results of enzyme inhibition *in vitro*, which suggested that the occurrence of adverse reactions should be strictly monitored when *L. aggregata* and diclofenac were used together in the clinic. Although the pharmacokinetic parameters of tolbutamide and warfarin *in vivo* may not be consistent with the *in vitro* results, it suggests that the coadministration of LDR with these two clinically common drugs can induce HDIs. In addition, the inconsistencies in pharmacokinetic behaviors of drugs *in vivo* and *in vitro* are common for the reason that the *in vivo* process of drugs is more complex. Further studies are needed to elucidate the effects of LDR on some drug transporters and whether LDR will affect the absorption process of CYP2C9 substrates *in vivo*.

## Figures and Tables

**Table 1:** The mass scan method parameters of diclofenac and chlorzoxazone (internal standard, IS).

Compounds	Q1 (m/z)	Q3 (m/z)	Dwell time (msec)	Cone (V)	Collision energy
Diclofenac	295.67	216.23^a^	0.044	20	20
		177.91^b^	0.044	20	30
Chlorzoxazone	167.78	131.88^a^	0.044	20	18
		75.85^b^	0.044	20	24

^a^Quantitative analysis. ^b^Qualitative analysis.

**Table 2:** Mass scan method parameters of tolbutamide, warfarin, and chlorzoxazone (internal standard, IS).

Compounds	Q1 (m/z)	Q3 (m/z)	DP (V)	EP (V)	CE (V)	CXP (V)
Tolbutamide	269.2	170.0	−55	−10	−26	−17
Warfarin	307.1	250.0	−30	−10	−31	−17
Chlorzoxazone	168.0	132.0	−90	−10	−28	−17

DP: declustering potential; EP: entrance potential; CE: collision energy; CXP: collision cell exit potential.

**Table 3:** Analytical curves, correlation coefficient (*r*), linear range, and LLOQ of diclofenac, tolbutamide, and warfarin

Compounds	Analytical curves	*r*	Linear range (*μ*g/mL)	LLOQ (*μ*g/mL)	RSD (%)	RE (%)
Diclofenac	y = 0.00210x + 0.00733	0.9998	0.020–5.0	0.020	10.91	0.0
Tolbutamide	y = 0.000252x + 0.0307	0.9973	0.200–100	0.200	3.47	5.0
Warfarin	y = 0.00271x + 0.0277	0.9977	0.050–20	0.050	4.24	3.2

**Table 4:** Intraday/interday accuracy, precision, recovery, and matrix effect of diclofenac, tolbutamide, and warfarin in rat plasma.

Compounds	Added concentration (*μ*g/mL)	Found concentration (*μ*g/mL)	Intraday RSD (%)	Interday RSD (%)	Accuracy RE (%)	Recovery (%)	Matrix effect (%)
Diclofenac	0.060	0.06 ± 0.00	5.76	5.32	1.1	60.01 ± 3.35	95.30 ± 5.33
	0.40	0.40 ± 0.01	3.52	3.49	2.9	58.83 ± 1.83	96.07 ± 3.00
	3.8	3.74 ± 0.05	1.46	4.64	0.5	63.02 ± 1.16	96.43 ± 1.78
Tolbutamide	0.600	0.61 ± 0.03	4.86	3.70	−4.7	63.58 ± 2.27	99.03 ± 3.64
	4.00	4.21 ± 0.03	0.82	3.39	6.6	63.83 ± 2.60	103.70 ± 0.02
	75.0	79.12 ± 1.41	1.79	3.58	10	64.27 ± 2.47	103.70 ± 0.02
Warfarin	0.15	0.15 ± 0.00	3.02	6.27	2.7	63.84 ± 1.89	92.30 ± 2.73
	1.5	1.64 ± 0.03	1.56	2.02	10.3	61.17 ± 0.84	103.09 ± 1.41
	15	14.20 ± 0.14	1.00	2.08	−6.9	71.84 ± 1.09	99.88 ± 1.52

**Table 5:** Stability of diclofenac, tolbutamide, and warfarin in rat plasma.

Compounds	Added concentration (*μ*g/mL)	Postpreparative stability		Short-term stability		Freeze-thaw stability		Long-term stability	
		Found concentration (*μ*g/mL)	RSD (%)	Found concentration (*μ*g/mL)	RSD (%)	Found concentration (*μ*g/mL)	RSD (%)	Found concentration (*μ*g/mL)	RSD (%)
Diclofenac	0.060	0.06 ± 0.00	3.7	0.06 ± 0.00	3.8	0.06 ± 0.00	6.6	0.06 ± 0.00	6.1
	3.8	3.63 ± 0.06	1.6	3.66 ± 0.08	2.1	3.88 ± 0.13	3.5	3.92 ± 0.11	2.9
Tolbutamide	0.600	0.58 ± 0.02	3.3	0.58 ± 0.03	4.8	0.56 ± 0.02	2.8	0.57 ± 0.01	2.2
	75.0	74.62 ± 0.84	1.1	75.78 ± 0.84	1.1	75.82 ± 1.51	2.0	75.15 ± 4.69	6.2
Warfarin	0.15	0.16 ± 0.01	4.0	0.16 ± 0.01	3.8	0.15 ± 0.00	2.8	0.16 ± 0.01	7.4
	15	13.90 ± 0.29	2.1	14.20 ± 0.14	1.0	14.68 ± 0.08	0.5	14.00 ± 0.37	2.6

**Table 6:** Pharmacokinetic parameters of diclofenac in the treated group and control group.

Pharmacokinetic parameters	Treated group	Control group
t1/2 (h)	0.87 ± 0.32	1.04 ± 0.37
T_max_ (h)	0.12 ± 0.06	0.11 ± 0.08
C_max_ (*μ*g/L)	1287.82 ± 454.16^*∗∗*^	258.66 ± 103.52
AUC0-t (mg/*L∗h*)	1509.59 ± 304.91^*∗∗*^	741.98 ± 215.78
AUC0-∞ (mg/*L∗h*)	1509.59 ± 304.91^*∗∗*^	741.99 ± 215.78
V_d_ (L/kg)	1.68 ± 0.42^*∗*^	4.66 ± 2.40
CL (L/h/kg)	1.44 ± 0.35^*∗∗*^	2.98 ± 0.76

Pharmacokinetic parameters were calculated by DAS 3.2.8 software. t1/2: elimination half-life; T_max_: the time of peak concentration; C_max_: the maximum concentration; AUC0-t, AUC0-∞: area under the plasma concentration-time profiles; V_d_: volume of distribution; CL: clearance.$\bar{x}\pm s,$*n*=6.^*∗*^*p* < 0.05; ^*∗∗*^*p* < 0.01; ^*∗∗∗*^*p* < 0.001, compared with the control group.

**Table 7:** Pharmacokinetic parameters of tolbutamide in the treated group and control group.

Pharmacokinetic parameters	Treated group	Control group
t1/2 (h)	5.33 ± 3.51	2.46 ± 0.11
T_max_ (h)	6.67 ± 3.01	6.00 ± 2.00
C_max_ (mg/L)	60.70 ± 10.70^*∗∗∗*^	82.72 ± 4.84
AUC0-t (mg/*L∗h*)	866.95 ± 147.51	923.84 ± 122.76
AUC0-∞ (mg/*L∗h*)	867.29 ± 147.80	923.84 ± 122.76
V_d_ (L/kg)	0.27 ± 0.15	0.12 ± 0.01
CL (L/h/kg)	0.04 ± 0.01	0.03 ± 0.00

Pharmacokinetic parameters were calculated by DAS 3.2.8 software. t1/2: elimination half-life; T_max_: the time of peak concentration; C_max_: the maximum concentration; AUC0-t, AUC0-∞: area under the plasma concentration-time profiles; V_d_: volume of distribution; CL: clearance.$\bar{x}\pm s,$*n*=6.^*∗*^*p* < 0.05; ^*∗∗*^*p* < 0.01; ^*∗∗∗*^*p* < 0.001, compared with the control group.

**Table 8:** Pharmacokinetic parameters of warfarin in the treated group and control group.

Pharmacokinetic parameters	Treated group	Control group
t1/2 (h)	18.46 ± 1.02	24.85 ± 17.93
T_max_ (h)	3.79 ± 2.46	24.85 ± 17.93
C_max_ (mg/L)	9.64 ± 0.70	9.60 ± 2.00
AUC0-t (mg/*L∗h*)	161.26 ± 16.84	277.59 ± 169.67
AUC0-∞ (mg/*L∗h*)	164.51 ± 16.91	329.51 ± 260.38
V_d_ (L/kg)	0.33 ± 0.04^*∗∗*^	0.23 ± 0.04
CL (L/h/kg)	0.01 ± 0.00	0.01 ± 0.00

Pharmacokinetic parameters were calculated by DAS 3.2.8 software. t1/2: elimination half-life; T_max_: the time of peak concentration; C_max_: the maximum concentration; AUC0-t, AUC0-∞: area under the plasma concentration-time profiles; V_d_: volume of distribution; CL: clearance.$\bar{x}\pm s,$*n*=6.^*∗*^*p* < 0.05; ^*∗∗*^*p* < 0.01; ^*∗∗∗*^*p* < 0.001, compared with the control group.

## Data Availability

The data used to support the findings of this study are included within the article.

## Abbreviations

AUC: Area under the plasma concentration-time profiles
CE: Collision energy
CL: Clearance
*C*_max_: Maximum concentration
CMC-Na: Sodium carboxyl methyl cellulose
CXP: Collision cell exit potential
DDIs: Drug-drug interactions
DP: Declustering potential
EDTA-Na2: Ethylenediaminetetraacetic acid disodium salt
EP: Entrance potential
HDIs: Herb-drug interactions
IS: Internal standard
LDR: Linderane
MRM: Multiple reaction monitoring
NADPH: Nicotinamide adenine dinucleotide phosphate
t1/2: Elimination half-life
*T*_max_: Time of peak concentration
Vd: Volume of distribution.
